# Phytopigment Alizarin Inhibits Multispecies Biofilm Development by *Cutibacterium acnes*, *Staphylococcus aureus*, and *Candida albicans*

**DOI:** 10.3390/pharmaceutics14051047

**Published:** 2022-05-12

**Authors:** Jin-Hyung Lee, Yong-Guy Kim, Sunyoung Park, Liangbin Hu, Jintae Lee

**Affiliations:** 1 School of Chemical Engineering, Yeungnam University, 280 Daehak-Ro, Gyeongsan 38541, Korea; jinhlee@ynu.ac.kr (J.-H.L.); yongguy7@ynu.ac.kr (Y.-G.K.); sunyong3142@ynu.ac.kr (S.P.); 2 School of Food & Biological Engineering, Shaanxi University of Science and Technology, Xi’an 710021, China; hulb@sust.edu.cn

**Keywords:** alizarin, antibiofilm, *Candida albicans*, *Cutibacterium acnes*, multispecies biofilms, *Staphylococcus aureus*

## Abstract

Acne vulgaris is a common chronic inflammatory skin disease involving *Cutibacterium acnes* with other skin commensals such as *Staphylococcus aureus* and *Candida albicans* in the anaerobic and lipid-rich conditions of pilosebaceous units. These microbes readily form multispecies biofilms that are tolerant of traditional antibiotics as well as host immune systems. The phytopigment alizarin was previously found to prevent biofilm formation by *S. aureus* and *C. albicans* strains under aerobic conditions. Hence, we hypothesized that alizarin might control *C. acnes* and multispecies biofilm development. We found that under anaerobic conditions, alizarin efficiently inhibited single biofilm formation and multispecies biofilm development by *C. acnes*, *S. aureus*, and *C. albicans* without inhibiting planktonic cell growth. Alizarin increased the hydrophilicities of *S. aureus* and *C. albicans* cells, decreased lipase production by *S. aureus*, diminished agglutination by *C. acnes,* and inhibited the aggregation of *C. albicans* cells. Furthermore, the co-administration of alizarin and antibiotics enhanced the antibiofilm efficacies of alizarin against *C. acnes*. A transcriptomic study showed that alizarin repressed the transcriptions of various biofilm-related genes such as lipase, hyaluronate lyase, adhesin/invasion-related, and virulence-related genes of *C. acnes*. Furthermore, alizarin at 100 µg/mL prevented *C. acnes* biofilm development on porcine skin. Our results show that alizarin inhibits multispecies biofilm development by acne-causing microbes and suggest it might be a useful agent for treating or preventing *C. acnes*-causing skin diseases.

## 1. Introduction

Acne vulgaris is an ordinary chronic skin disease that blocks and/or inflames hair follicles and their sebaceous glands, and the microbial colonization of pilosebaceous units by a Gram-positive anaerobic bacterium, *Cutibacterium acnes* (formerly *Propionibacterium* *acnes*) is a major pathogenic factor [1]. *C. acnes* produces several virulence factors, such as lipases, fatty acid isomerase, hemolysins, and porphyrins, that can deteriorate host tissues and promote perifollicular inflammation [1,2]. Furthermore, *C. acnes* readily forms biofilms and is often observed in multispecies biofilms with skin-colonizing microorganisms such as *Staphylococcus* *aureus* and *Candida albicans* [3,4,5].

Microbial biofilms are communities of microorganisms that adhere to surfaces or interfaces using self-manufactured extracellular polymeric substances (EPS). Biofilms exhibit a reduced sensitivity to traditional antimicrobial agents and host immune systems, and thus, provide bacterial resistance in chronic infections [6]. Hence, it is important to discover novel compounds able to prevent biofilm formation.

Phytochemicals represent an important resource of antibiofilm molecules against various pathogens. Previously, the phytopigment alizarin was found to efficiently suppress single biofilm formation by *S. aureus* [7] and *C. albicans* [8] under aerobic conditions for one day. Alizarin (also known as 1,2-dihydroxyanthraquinone) is a natural red dye and is used in studies on bone growth and has recently shown greater cytotoxicity to bone tumor cells than normal cells [9]. Hence, the current study was designed to analyze the effect of alizarin on biofilm formation by single and multiple organisms including acne-related *C. acnes*, *S. aureus*, and *C. albicans* in the mixed culture media under anaerobic conditions for 7 days.

To investigate how alizarin influences biofilm formation by *C. acnes*, we used a three species biofilm model comprised of *C. acnes*, *S. aureus*, and *C. albicans*, scanning electron microscopy (SEM), confocal laser scanning microscopy (CLSM), and transcriptomic analysis, and assessed cell surface hydrophilicity, lipase production, cell agglutination, and cell aggregation. In addition, the antibiofilm efficacy of alizarin was investigated and compared with three antibiotics (benzoyl peroxide, gentamycin, or ciprofloxacin) and in a porcine skin model.

## 2. Materials and Methods

### 2.1. Strains and Chemicals

The strains used were as follows; human facial acne derived *C. acnes* ATCC 6919, dental plaque derived *C. acnes* KCCM 42791, MSSA (methicillin-sensitive *S. aureus*) ATCC 6538, and a fluconazole-resistant *C. albicans* DAY185. Strains were obtained from the ATCC (American Type Culture Collection, Manassas, VA, USA) and the KCCM (Korean Culture Center for Microorganisms, Seoul, Korea). *C. acnes* strains were cultivated using RCM (Reinforced Clostridium Media)-agar plates and in liquid RCM at 37 °C in anaerobic pouches (BD GasPak™ EZ Gas Generating Anaerobic Pouch Systems; Fisher Scientific, Pittsburgh, PA, USA). *S. aureus* was cultivated on LB (Lysogeny Broth)-agar plates and in LB liquid medium at 37 °C, and *C. albicans* was cultivated on PDB (Potato Dextrose Broth)-agar plates and in PDB liquid medium at 37 °C in anaerobic pouches.

Alizarin, dimethyl sulfoxide (DMSO), crystal violet, glutaraldehyde, formaldehyde, p-nitrophenyl palmitate, isopropyl alcohol, gummi arabicum, sodium deoxycholate, sodium carbonate, disodium phosphate, *Saccharomyces cerevisiae*, and hexadecane were obtained from Sigma-Aldrich (St. Louis, MO, USA), TCI (Tokyo Chemical Industry Co., Ltd., Tokyo, Japan) or Combi-Blocks, Inc. (San Diego, CA, USA). Dimethyl sulfoxide (DMSO) was employed to liquify alizarin, and 0.1% (*v*/*v*) DMSO was employed as the control; at this concentration DMSO did not show any effect on bacterial growth or biofilm formation.

### 2.2. MIC Determinations and Planktonic Cell Growth Measurements

To investigate MIC and the effect of alizarin on the planktonic cell growth of *C. acnes*, 7-day cultures were inoculated (dilution 1:50) into fresh RCM liquid medium and incubated with different concentrations of alizarin (0, 1, 2, 5, 10, 20, 50, 100, 200, or 400 μg/mL) in 96-well plates (SPL Life Sciences, Pocheon, Korea) for 7 days at 37 °C in anaerobic pouches, and cell growth was then measured at 620 nm by a Multiskan EX microplate reader (Thermo Fisher Scientific, Waltham, MA, USA) [10]. Four independent samples were analyzed for each experiment.

### 2.3. Biofilm Formation Suppression Assay

Biofilm development was measured using 0.1% crystal violet, as previously described [11]. Briefly, a 7-day culture of *C. acnes* was diluted in a 1:50 ratio with fresh RCM liquid medium (CFU ~ 2 × 10^6^ cells/mL), added in a 96-well plate with or without alizarin, and incubated at 37 °C in anaerobic pouches for 7 days (total 300 µL/well). Free floating cells were then removed, and wells were rinsed three times with water. Attached biofilm cells in wells were stained with 300 μL of crystal violet for 20 min, rinsed three times with distilled water (to remove excess crystal violet), and crystal violet stains were then extracted with 95% ethanol. Absorbances (surrogates of biofilm formation) were measured at 570 nm by a Multiskan EX microplate reader. Results were presented as the averaged values of at least 6 replicate wells of 2 independent cultures. Biofilm percentages were calculated by expressing absorbances in the presence of alizarin as percentages of untreated control absorbances. Alizarin inhibitory effects on cell growth and biofilm formation were compared with those of benzoyl peroxide (1, 2, 5, 10, 20, 50, or 100 µg/mL) and the antibiotics gentamycin (1, 2, 5, 10, 20, 50, or 100 µg/mL) or ciprofloxacin (1, 2, 5, 10, 20, 50, or 100 µg/mL).

To assess *S. aureus* and *C. albicans* biofilm formation, cells were inoculated at dilutions of 1:100 and 1:50 into fresh LB and PDB liquid media, respectively, and then incubated under anaerobic conditions at 37 °C for 7 days. Biofilm development was investigated as described for *C. acnes* above. All biofilm experiments were tested at least four independent samples for each experiment.

### 2.4. Biofilm Observations by Live Imaging Microscopy and SEM

To analyze biofilms produced by *C. acnes*, *S. aureus*, or *C. albicans* or *C. acnes*/*S. aureus*/*C. albicans* with or without alizarin (0, 2, 10, or 50 μg/mL), cells were incubated under anaerobic conditions at 37 °C for 7 days. After incubation, planktonic cells were discarded and rinsed three times with distilled water and biofilms were observed by live imaging microscopy (iRiS^TM^ Digital Cell Imaging System) at different magnifications. Biofilm pictures were regenerated as color-coded 2D/3D images by ImageJ (https://imagej.nih.gov/ij/index.html). Single *C. acnes* and multispecies biofilm development on nylon filter membranes were also examined by SEM, as previously described [12]. Briefly, 0.4 × 0.4 cm size of nylon filter membrane (Merck Millipore, Burlington, USA) pieces were placed in each well of 96-well plates having *C. acnes* or *C. acnes*, *S. aureus*, and *C. albicans* and incubated with or without alizarin (0, 2, 10, or 50 μg/mL) in anaerobic pouches at 37 °C for 7 days. Biofilms on membranes were fixed by treatment of glutaraldehyde (2.5%) and formaldehyde (2%) for 16 h, post-fixed with OsO_4_ solution, and samples were dehydrated by treatment of an ethanol series (50, 70, 80, 90, 95, and 100%) followed by 3-methylbutyl acetate. After drying samples using a critical point dryer (HCP-2, Hitachi, Tokyo, Japan), biofilm cells on nylon filter membranes were examined and imaged by a S-4200 or S-4800 SEM (scanning electron microscope) (Hitachi, Tokyo, Japan). For both live imaging analysis and SEM, two independent samples were analyzed for each experiment.

### 2.5. Biofilm Observations by Confocal Microscopy

Single strain or multispecies biofilms of *C. acnes*, *S. aureus*, and *C. albicans* were produced in 96-well plates (with or without alizarin) at 37 °C for 7 days under anaerobic conditions. Free floating cells were then discarded by rinsing with water three times, and biofilm cells attached on the surface of wells were stained with CFDA-SE (carboxyfluorescein diacetate succinimidyl ester) (Invitrogen, Molecular Probes, Inc, Eugene, OR, USA). The bottom of each well was then visualized by a 488 nm Ar laser (emission 500 to 550 nm) using a confocal laser microscope Nikon Eclipse Ti (Nikon, Tokyo, Japan). COMSTAT software [13] was employed to determine biovolumes (μm^3^/μm^2^), mean biofilm thicknesses (μm), substratum coverages (%), and roughness coefficients. Two independent samples were analyzed for each experiment and more than 12 random spots were observed.

### 2.6. Assay of Multispecies Biofilm Development by C. acnes, S. aureus, and C. albicans

In order to quantify multispecies biofilm formation, we used a previously described method [14]. Briefly, *C. acnes*, *S. aureus* cells, and *C. albicans* cells were co-inoculated into mixed culture medium (RCM/LB/PDB = 1:1:1) in 96-well plates and the final inocula were *C. acnes* (12 ± 4 × 10^5^ CFU/mL), *S. aureus* cells (40 ± 4 × 10^6^ CFU/mL), and *C. albicans* (46 ± 3 × 10^3^ CFU/mL). The mixed culture was incubated with or without alizarin (0, 2, 10, or 50 μg/mL) under anaerobic conditions at 37 °C for 7 days. After incubation, biofilm development was investigated as described above. Two independent samples were analyzed.

### 2.7. Cell Agglutination as Determined by EPS Production

To quantify EPS production, we used a previously described method [15]. 0.5 mL cultures of *C. acnes, S. aureus,* or *C. albicans* were treated or not with alizarin (0, 1, 2, 5, 10, 20, 50, or 100 μg/mL) at 37 °C for 7 days under anaerobic conditions and then adjusted to ~0.5 at OD_600_. Cultures were added to 1.5 mL of phosphate-buffered saline (PBS) and 0.5 mL of 2% *Saccharomyces cerevisiae* solution (*w*/*v* in PBS) and vigorously vortexed for 30 sec. Initial turbidity at 600 nm was measured by an Optizen 2120 UV spectrophotometer (Mecasys Co. Ltd., Daejeon, Korea). These mixtures were then incubated for ~30 min at room temperature and the semi-clear upper phases that separated out were transferred to a 96-well plate. Absorbances were measured at 620 nm by a Multiskan EX microplate reader. Two independent samples were analyzed.

### 2.8. Cell Surface Hydrophobicity

The effects of alizarin (0, 1, 2, 5, 10, 20, 50, or 100 µg/mL) on the surface hydrophobicities of *C. acnes*, *S. aureus*, and *C. albicans* were assessed using the previous assay [16]. Briefly, *C. acnes*, *S. aureus*, or *C. albicans* were incubated with or without alizarin for 7 days at 37 °C under anaerobic conditions, collected by centrifugation at 8000× *g* for 5 min, and resuspended in PBS. Cells were resuspended in PBS at an OD_600_ ~ 0.5, and then 3 mL of toluene was added to 3 mL aliquots, mixed by vortexing for 90 s, and incubated at 25 °C for 8 h when the toluene and aqueous phases completely separated. Two independent samples were analyzed. The turbidity values of aqueous phases at OD_600_ were measured and cell hydrophobicities (hydrophobicity indices) were calculated using the formula derived by Sivasankar et al. [17], as follows:

[1 − (OD_600_ after phase separation/OD_600_ before adding toluene)] × 100

### 2.9. Lipase Production Assay

To investigate the effect of alizarin on extracellular lipase productions by *C. acnes*, *S. aureus*, or *C. albicans,* they were diluted into RCM, LB, or PDB at 1:50, 1:100, and 1:50, respectively, and incubated under anaerobic conditions for 7 days at 37 °C with or without alizarin (0, 1, 2, 5, 10, 20, 50, or 100 µg/mL). Supernatants were then collected by centrifugation at 10,000× *g* for 15 min, and 0.1 mL aliquots of supernatants were mixed with 900 µL of substrate buffer (1 part (vol.) of buffer A having 3 mg/mL of *p*-nitrophenyl palmitate in 2-propanol + 9 parts (vol.) of buffer B containing 1 mg/mL of gummi arabicum and 2 mg/mL sodium deoxycholate in 0.05 M disodium hydrogen phosphate buffer (pH 8.0)) at 25 °C for 30 min under dark conditions. The lipase reaction was then stopped by adding 0.1 mL of 1 M sodium carbonate and samples were centrifuged at 10,000× *g* for 15 min. Absorbances of supernatants were measured at 405 nm as previously described [15]. Two independent samples were analyzed.

### 2.10. Cell Aggregation in Filamentous Growth Promoting Liquid Media

Cell aggregation and filamentous growth were investigated as previously described [18], *C. albicans* cells were inoculated in 1.5 mL of hyphae promoting RPMI-1640 medium supplemented with 10% fetal bovine serum at a cell density of 10^5^ CFU/mL in 1.6 mL tubes with or without alizarin (2, 10, or 50 μg/mL) and incubated under anaerobic conditions at 37 °C for 24 h. After incubation, aggregated cells and filamentous growths were analyzed using the iRiS^TM^ Digital Cell Imaging System (Logos Biosystems, Anyang, Korea) under bright field at 4× and 10× magnifications. At least four independent experiments were performed.

### 2.11. Transcriptomic Studies by Quantitative Reverse Transcriptase Real-Time PCR (qRT-PCR)

For transcriptomic expression analyses, *C. acnes* cells (~23 ± 3 × 10^5^ CFU/mL, OD_600_ = 0.05) were diluted in 15 mL of fresh RCM broth in 15-mL sterile conical tubes and incubated without agitation in anaerobic pouches for 7 days at 37 °C; alizarin (100 µg/mL) or DMSO (the control) added at the beginning of incubation. To avoid RNA degradation, RNase inhibiting agent (RNAlater, Thermo fisher Scientific, Watham, MA, USA) was added to cells just before harvest. Total RNA was isolated and purified by RNeasy Mini Kits (Qiagen, Valencia, CA, USA).

qRT-PCR reactions were performed to analyze the transcriptional expressions of biofilm- and virulence-related genes. The sequence of primers used for qRT-PCR are provided in Table S1. The expression of *16s rRNA* (the housekeeping gene) was not changed by alizarin. qRT-PCR was performed as previously described [12] using SYBR Green master mix (Applied Biosystems, Foster City, CA, USA) and the ABI StepOne Real-Time PCR System (Applied Biosystems). qRT-PCR reactions were conducted in quadruplicate using least two independent cultures.

### 2.12. Biofilm Inhibition Analysis on Porcine Skin

The assay used was a modification of the method devised by Yang et al. [19]. Briefly, freshly frozen porcine skin was purchased from the Korea Federation of Livestock Cooperatives (Seoul, Korea) and stored at –80 °C until required. Skin was sterilized before use by immersing 0.7 × 0.7 cm pieces sequentially in 70% ethanol and 10% bleach solution for 30 min each. Pieces were then washed with autoclaved water for 3 × 10 min. *C. acnes* cells were inoculated in RCM, added to a 12-well plate containing skin pieces, and incubated with or without alizarin (0, 20, 50, and 100 µg/mL) under anaerobic conditions for 7 days at 37 °C. SEM was performed as described above. Two independent samples were analyzed.

### 2.13. Statistical Analysis

The statistical analysis was performed by one-way ANOVA followed by Dunnett’s test in SPSS Ver. 23 (Chicago, IL, USA). Results are presented as means ± SDs, and *p* values of <0.05 were considered significant indicated with asterisks.

## 3. Results

### 3.1. Antibiofilm Activity of Alizarin against C. acnes

**Figure 1:**
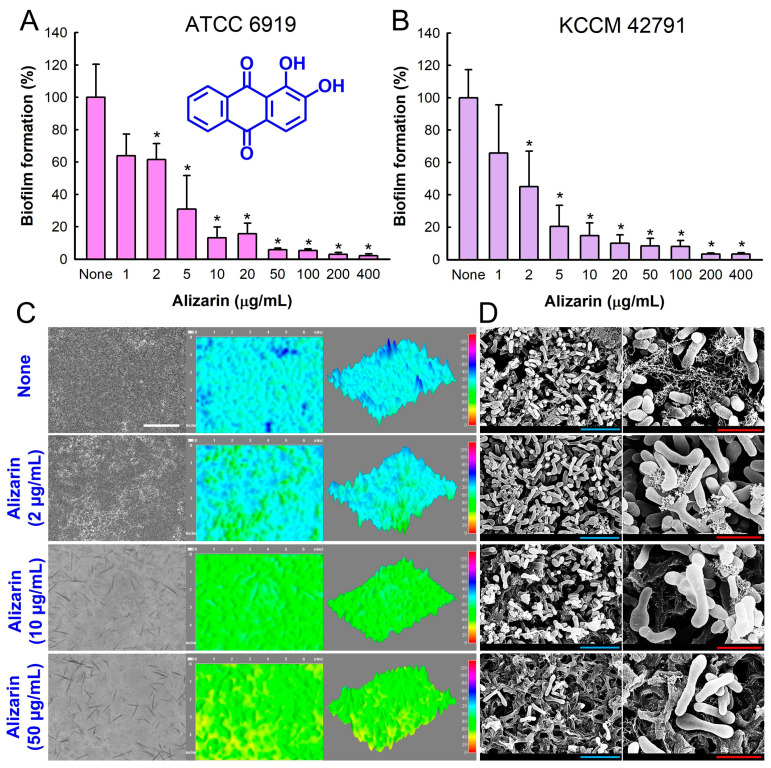
Alizarin inhibited biofilm formation by *C. acnes*. The antibiofilm activity of alizarin on *C. acnes* ATCC 6919 (**A**) and KCCM 42791 (**B**) was investigated in 96-well polystyrene plates for 7 days under anaerobic conditions. Chemical structure of alizarin is shown. (**A**) Regenerated *C. acnes* ATCC 6919 biofilm color-coded 2D and 3D images after culture with alizarin. (**C**) SEM images of *C. acnes* ATCC 6919 biofilms developed in the presence or absence of alizarin. (**D**) White, blue, and red scale bars indicate 100, 5, and 2 μm, respectively. None: non-treated control. Error bars represent standard deviations. * *p* < 0.05 vs. non-treated controls.

The antibiofilm activity of alizarin was initially analyzed against *C. acnes* ATCC 6919 in 96-well polystyrene plates for 7 days under anaerobic conditions. Alizarin dose-dependently prevented biofilm formation by *C. acnes* (Figure 1A); for example, alizarin at 10 and 100 µg/mL inhibited *C. acnes* biofilm formation by 86% and 94%, respectively. The trend of antibiofilm activity of alizarin against another *C. acnes* strain KCCM 42,791 isolated from dental plaque was similar (Figure 1B). Hence, we focused on *C. acnes* ATCC 6919 isolated from human facial acne in further experiments.

An MIC assay of alizarin against *C. acnes* planktonic cells revealed it had an MIC of >400 µg/mL. However, due to the intense red color of alizarin, its MIC was difficult to measure optically. Hence, CFUs were also measured and alizarin was found not to reduce CFUs at concentrations of ≤400 µg/mL (~115 × 10^8^ CFU/mL). The results indicated that at subinhibitory concentrations (2–400 µg/mL), alizarin inhibited *C. acnes* biofilm formation without affecting free floating cell growth. Furthermore, these results suggested that alizarin might be less prone to the development of drug tolerance.

Biofilm growth on polystyrene surfaces was investigated by bright-field microscopy as 2-D and 3-D LUT mesh plots. Alizarin at 2–50 µg/mL dose-dependently reduced biofilm formation (Figure 1C), and SEMs showed that alizarin reduced EPS production and *C. acnes* cell densities in biofilms (Figure 1D). Interestingly, alizarin treatment also increased cell length in biofilms, indicating that it inhibited cell division. Specifically, the sizes of *C. acnes* biofilm cells were 234% (3.8 ± 0.6 μm) and 242% (4.0 ± 0.5 μm) greater after alizarin treatment for 7 days at 2 or 10 µg/mL alizarin, respectively, than those of non-treated controls (None, 1.25 ± 0.4 μm).

### 3.2. Alizarin Inhibited Biofilm Development by S. aureus and C. albicans under Anaerobic Conditions

**Figure 2:**
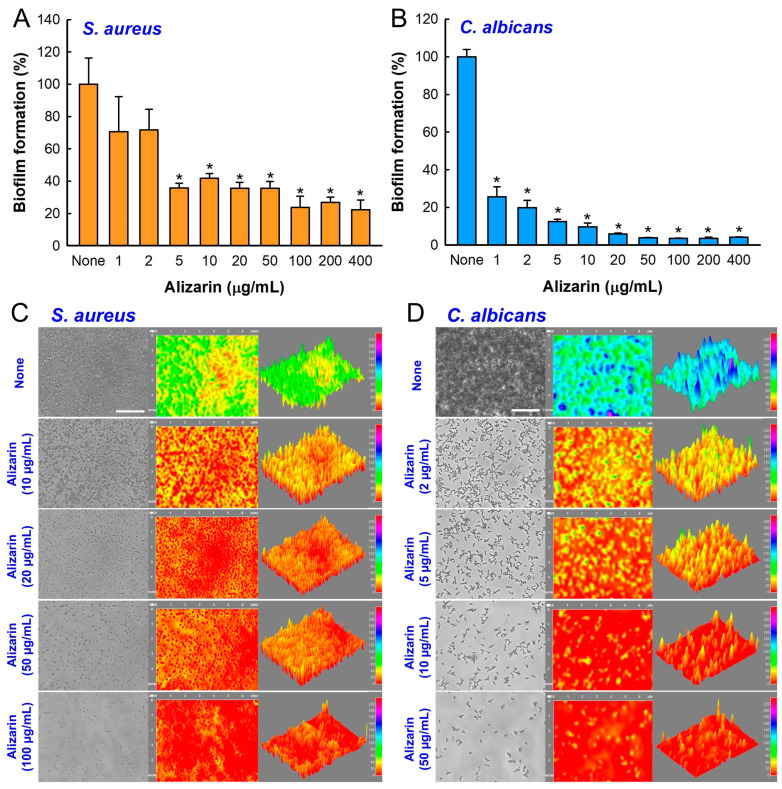
Inhibitory effect of alizarin on the biofilm development by *S. aureus* and *C. albicans* under anaerobic conditions. Biofilm developments by *S. aureus* (**A**) and *C. albicans* (**B**) were assessed after incubation for 7 days in 96-well plates under anaerobic conditions. Error bars represent standard deviations. * *p* < 0.05 vs. non-treated controls (None). Color-coded 2D and 3D images of biofilms after culturing *S. aureus* (**C**) or *C. albicans* (**D**) in the presence of alizarin for 7 days. White scale bars represent 100 μm.

The inhibitory effect of alizarin on the biofilm development by *S. aureus* and *C. albicans* were investigated under anaerobic conditions for 7 days because *S. aureus* and *C. albicans* are facultative organisms that can grow under aerobic and anaerobic conditions. Alizarin dose-dependently reduced biofilm development by *S. aureus* (Figure 2A) and *C. albicans* (Figure 2B). For example, alizarin treatment for 7 days at 10 µg/mL inhibited *S. aureus* biofilm development by 58% and *C. albicans* biofilm formation by 90%. These results are slightly lower than those previously reported for alizarin against *S. aureus* [7] and *C. albicans* [8] under aerobic conditions, which were ascribed to the longer incubation (7 days vs. 1 day) and anaerobic conditions used in the present study. Microscopic observations of biofilms confirmed that alizarin dose-dependently inhibited *S. aureus* and *C. albicans* biofilm development (Figure 2C,D).

### 3.3. Alizarin Prevented Multispecies Biofilm Development by C. acnes, S. aureus, and C. albicans

**Figure 3:**
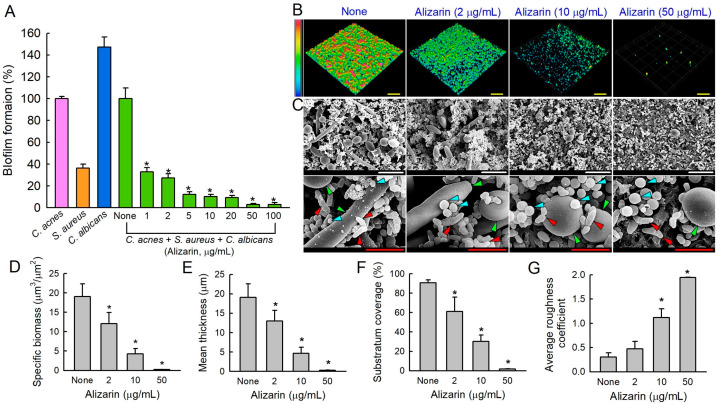
Alizarin inhibited multispecies biofilms. The antibiofilm effects of alizarin on *C. acnes/S. aureus/C. albicans* biofilms (**A**) after 7-day culture under anaerobic conditions. * *p* < 0.05 vs. non-treated controls (None). CLSM images of biofilm inhibition (**B**) and COMSTAT analysis of CLSM images (**D**–**G**). Yellow scale bars represent 100 µm. SEM images of polymicrobial biofilms developed in the presence or absence of alizarin (**C**). White and red scale bars indicate 10 and 3 µm, respectively. Red, blue, and green triangles represent *C. acnes*, *S. aureus*, and *C. albicans*, respectively. None; non-treated control.

We developed a multispecies biofilm model of *C. acnes*, *S. aureus*, and *C. albicans* using a Reinforced Clostridium Media (RCM), Lysogeny Broth (LB), and Potato Dextrose Broth (PDB) mixed medium (1:1:1) and anaerobic conditions [20]. Hence, the antibiofilm efficacy of alizarin was tested on the three species biofilm model. In the mixed medium, fungal *C. albicans* formed the strongest biofilms followed by *C. acnes* and *S. aureus* (Figure 3A). Alizarin dose-dependently reduced multispecies biofilm formation (Figure 3A), for example, alizarin at 1 or 10 µg/mL reduced biofilm development by more than 65% and 89%, respectively, after the 7-day culture.

CLSM and SEM confirmed multispecies biofilm reduction by alizarin. In the absence of alizarin, ~20 µm thick multispecies biofilms formed with almost 100% surface coverage, while in the presence of alizarin at 10 or 50 µg/mL, biofilm densities and thicknesses were dramatically reduced (Figure 3B). SEM was used to confirm the presence of each species in these mixed biofilms (Figure 3C). In non-treated controls, *C. albicans* produced large pseudohyphae and yeast cells and round *S. aureus* cells and rod-type *C. acnes* cells appeared. Interestingly, at concentrations >10 µg/mL, alizarin reduced EPS production and inhibited the adhesion of most *C. albicans* cells, while few *C. acnes* and *S. aureus* cells were detected.

COMSTAT analysis revealed more detail of biofilm structural changes (Figure 3D–G). Non-treated *C. acnes* produced dense biofilms (thickness 19 µm and 90% surface coverage), whereas in the presence of alizarin at 2, 10, or 50 µg/mL, biofilm densities and thicknesses were significantly reduced (Figure 3B). More specifically, alizarin at 50 µg/mL reduced biofilm biomasses, thicknesses, and substrate coverages by >98% vs. untreated controls and increased biofilm roughness.

### 3.4. Alizarin Increased the Antibiofilm Activities of Antibiotics against C. acnes

The antibiofilm activities of alizarin and three antibiotics, namely, benzoyl peroxide, gentamycin, or ciprofloxacin were investigated. While alizarin alone at 2 µg/mL inhibited *C. acnes* biofilm formation by 40% (Figure 1A), antibiofilm activity was significantly enhanced when it was used in combination with an antibiotic. For example, benzoyl peroxide at 100 μg/mL and alizarin at 2 µg/mL reduced biofilm development by 95% (Figure S1A), and gentamycin at 5 μg/mL and alizarin at 2 µg/mL reduced biofilm formation by 80% (Figure S1B). However, the alizarin (2 µg/mL) and ciprofloxacin (0.25 μg/mL) was less effective (Figure S1C). These results suggest the antibiofilm mechanisms of alizarin and the three antibiotics differ and that alizarin could be used as an antibiotic adjuvant.

### 3.5. Effects of Alizarin on Cell Surface Hydrophilicity, Lipase Production, and Cell Agglutination

**Figure 4:**
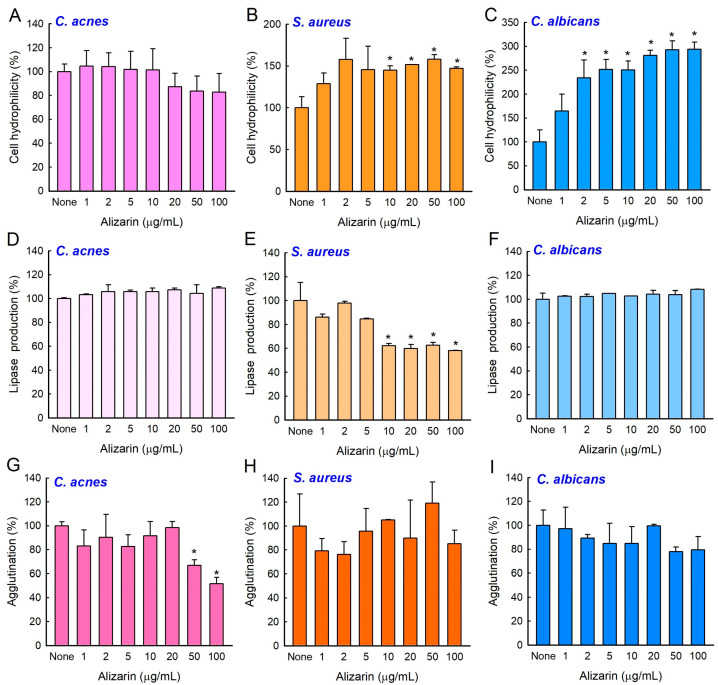
Effects of alizarin on virulence factors. Cell surface hydrophobicities of *C. acnes*, (**A**) *S. aureus*, (**B**) and *C. albicans*, (**C**) extracellular lipase production by *C. acnes*, (**D**) *S. aureus*, (**E**) and *C. albicans*, (**F**) cell agglutination by *C. acnes*, (**G**) *S. aureus*, (**H**) and *C. albicans*, (**I**) Error bars indicate standard deviations. * *p* < 0.05 vs. non-treated controls (None).

To understand how alizarin affects biofilm formation, surface hydrophilicity, lipase production, and the agglutination of *C. acnes*, *S. aureus*, or *C. albicans* were investigated. Cell surface hydrophobicity (CSH) was determined by assessing cell attachment to abiotic and biotic surfaces [21]. While alizarin did not affect *C. acnes* CSH (Figure 4A), it significantly increased *S. aureus* (Figure 4B) and *C. albicans* (Figure 4C) CSHs at 10–100 µg/mL. Bacterial lipases are associated with virulence and aid biofilm formation [2], and the activity of extracellular lipases were inhibited by alizarin at 10–100 µg/mL in *S. aureus* but not in *C. acnes* or *C. albicans* (Figure 4D–F). Cell agglutination is associated with biofilm formation [22] and was slightly inhibited by alizarin at 50–100 µg/mL in *C. acnes* but not in *S. aureus* or *C. albicans* (Figure 4G–I). These results suggest that different mechanisms underlie the activities of alizarin in these three microbes.

### 3.6. Alizarin Inhibited Cell Aggregation by C. albicans

**Figure 5:**
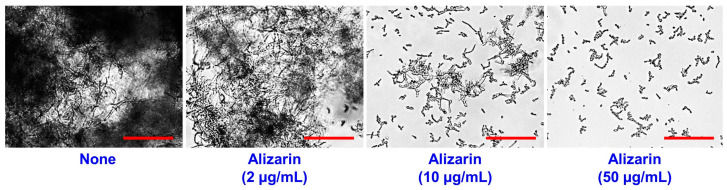
Effects of alizarin on *C. albicans* aggregation and filamentous growth. Cell aggregation was investigated in RPMI medium having 10% fetal bovine serum. Scale bars represent 100 μm.

The transition of yeast cells to hyphal cells and cell aggregation are prerequisites of *C. albicans* biofilm development [23]. Previously, it was found that alizarin suppressed hyphae formation in *C. albicans* under aerobic conditions [8]. In the present study, cell aggregation was assayed based on microscopic observations. For untreated *C. albicans*, hyphal entanglement appeared to cause large cell aggregations after 24 h under anaerobic conditions, and alizarin treatments at 10–50 µg/mL substantially inhibited filamentous (hyphal) growth and these aggregations (Figure 5), which confirmed that alizarin potently inhibited *C. albicans* biofilm development by preventing hyphal formation and cell aggregation under anaerobic conditions.

### 3.7. Alizarin Altered Gene Expressions in C. acnes

**Figure 6:**
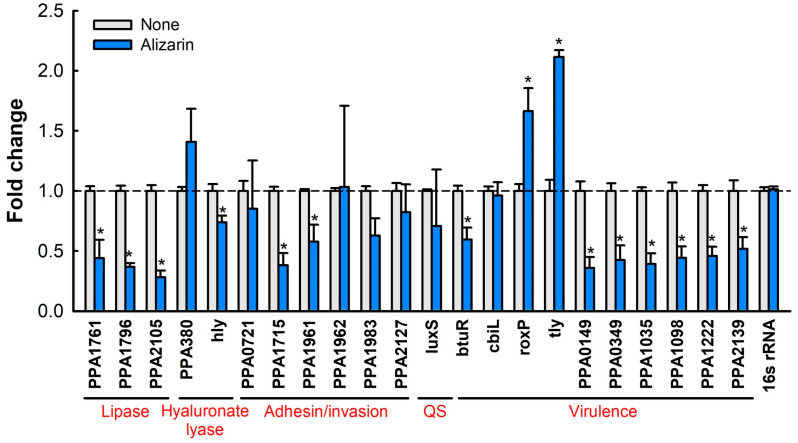
Relative gene expression profiles in *C. acnes* cells treated with alizarin. *C. acnes* cells were treated with alizarin at 10 µg/mL for 7 days without shaking. Fold changes indicate transcriptional differences observed in treated vs. untreated *C. acnes* (None) using qRT-PCR. QS indicates quorum sensing. Detailed information of genes is present in Table S1. The experiment was assessed in duplicate (six qRT-PCR reactions were assessed per gene). * *p* < 0.05 vs. non-treated controls (None).

To understand the action mechanism of alizarin in *C. acnes*, the differential expressions of 22 biofilm- and virulence-related genes were examined by real-time qRT-PCR. Alizarin was found to modulate the transcriptional expressions of several lipase genes, hyaluronate lyase genes, and virulence-related genes (Figure 6) but not to alter the expression of the housekeeping gene (*16s rRNA*). Importantly, the expressions of lipase (PPA1761, PPA1796, and PPA2105), hyaluronate lyase (*hly*), adhesin/invasion- (PPA1715 and PPA1961) and virulence-related genes (*btuR*, PPA0149, PPA0349, PPA1035, PPA1098, PPA1222, and PPA2139) were downregulated while the expressions of the antioxidant gene (*roxP*) and hemolysin (*tly*) were upregulated by alizarin at 10 µg/mL.

### 3.8. Alizarin Eradicated C. acnes on Porcine Skin

**Figure 7:**
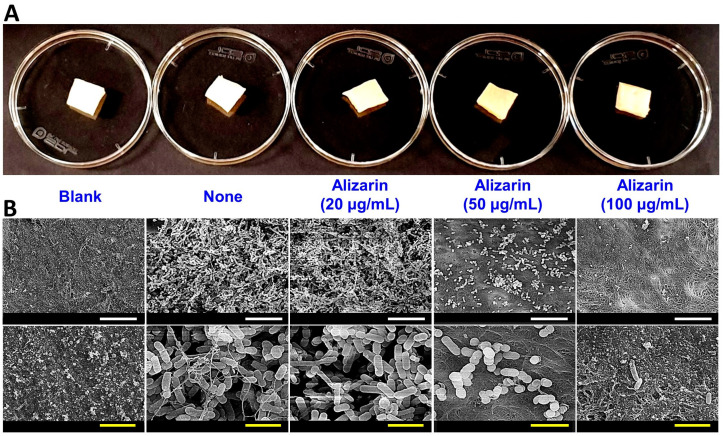
Effects of alizarin on biofilm development by *C. acnes* on porcine skin. Antibiofilm activities of alizarin against biofilm formation on porcine skin (**A**) were assessed by SEM (**B**) after 7-day culture under anaerobic conditions. White and yellow scale bars indicate 20 and 4.29 µm, respectively (**B**).

Since *C. acnes* is a common inhabitant of animal skin, we assessed the ability of alizarin to prevent biofilm development and the cell growth of *C. acnes* on porcine skin (Figure 7A). SEM was used to examine *C. acnes* biofilms (Figure 7B). Some background microflora was observed on blank controls without *C. acnes* inoculation, while mixed microorganisms (*C. acnes* and existing microbial flora) were observed on *C. acnes* untreated and alizarin treated skin. Alizarin at 20 µg/mL inhibited the production of EPS by *C. acnes* and alizarin at 20 or 50 µg/mL, eliminated bacterial biofilms, and markedly reduced background flora.

## 4. Discussion

**Figure 8:**
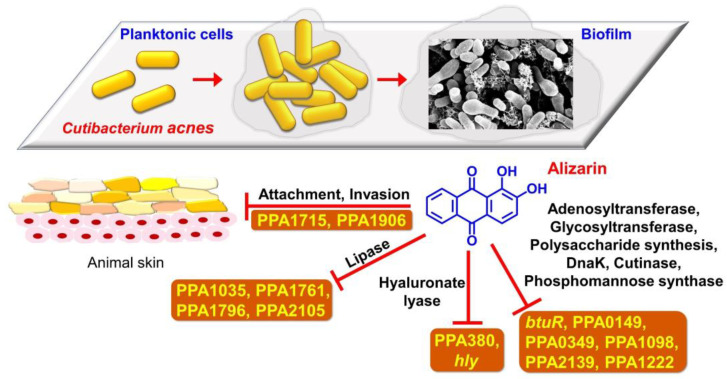
Diagram of the putative mechanism of alizarin in *C. acnes*.

Acne vulgaris is an ordinary chronic skin disease in young people often aggravated by multispecies biofilm development by microorganisms such as *C. acnes*, *S. aureus*, and *C. albicans* [24,25]. The current study demonstrates that the phytopigment alizarin has antibiofilm activity against each species of *C. acnes*, *S. aureus*, or fungal *C. albicans* as well as multispecies under anaerobic conditions, and partially reveals the mechanism involved (Figure 8).

Anthraquinones are a class of naturally occurring phenolics with the 9,10-anthraquinone skeleton (Figure 1A) and are often found in plants such as *Rubia* sp. [26,27]. Anthraquinones are widely used medicinally and industrially as antimalarials, laxatives, dyes, and antimicrobial agents [28]. Several anthraquinone derivatives, such as alizarin, chrysazin, emodin, purpurin, and quinalizarin, have been shown to have antibiofilm activity against several microbes, such as *Streptococcus mutans* [29], *S. aureus* [7,30], and *C. albicans* [8]. Particularly, alizarin has been reported to efficiently inhibit biofilm development by *S. aureus* [7,30] and *C. albicans* [8] under aerobic conditions. In addition, alizarin-conjugated graphene oxide composites were used to inhibit *C. albicans* biofilm formation, and alizarin exhibited in vitro anticancer activity and was substantially less toxic to normal cells [9].

Biofilm development mechanisms of Gram-negative bacteria, Gram-positive bacteria, yeast, and even among species, differ. *C. acnes* and *S. aureus* are Gram-positive pathogens and *C. albicans* is a pathogenic yeast, and as was expected, these three microbes responded differently to alizarin in terms of cell surface hydrophilicity, lipase production, and agglutination (Figure 4). In *S. aureus*, alizarin was found to suppress the effect of calcium and the expressions of the α-hemolysin *hla* gene, biofilm-related genes (*psmα*, *rbf*, and *spa*), and modulated the expressions of *cid*/*lrg* genes (the holin/antiholin system) [7]. Furthermore, it has been well demonstrated that yeast to hyphal cell transition is closely associated with biofilm development and the pathogenesis of *C. albicans* [31], and alizarin was found to inhibit hyphal formation by downregulating the expressions of various hypha-specific and biofilm-related genes (*ALS3*, *ECE1*, *ECE2*, and *RBT1*) [8]. The present study also confirms that alizarin inhibits *C. albicans* cell aggregation and hyphal growth (Figure 5).

Hydrophobicity plays an important role in biofilm development as often hydrophobic microbes prefer hydrophobic surfaces [32,33,34]. Interestingly, alizarin increased the hydrophilicities of *S. aureus* and *C. albicans* (Figure 4B,C), and thus, reduced cell attachment to the hydrophobic surfaces of plastic wells. Current results match with the previous report, in which increased cellular hydrophilicity decreased biofilm development by *C. acnes* [20] and *S. aureus* [35].

*C. acnes* expresses multiple biofilm- and virulence-related genes such as lipases, hyaluronate lyase, adhesin/invasion, and toxins. Our qRT-PCR study showed that alizarin suppressed the expressions of lipase genes (PPA1761, PPA1796, and PPA2105), hyaluronate lyase (*hly*), adhesion and invasion-related genes (PPA1715 and PPA1961), and virulence-related genes (*btuR*, PPA0149, PPA0349, PPA1035, PPA1098, PPA1222, and PPA2139) genes but upregulated the expressions of the antioxidant gene (*roxP*) and hemolysin (*tly*) (Figure 6).

*C. acnes* secretes various lipases that degenerate sebum lipids [1] and secreted lipases have been associated with its biofilm formation and associated inflammation [36]. Furthermore, alizarin also suppressed the expression of hyaluronate lyase (*hly*; a putative virulence factor), which is capable of destroying the epidermal extracellular matrix and might promote inflammation spread [2]. Interestingly, it was recently reported that 3,3′-diindolylmethane (an antibiofilm agent) downregulated the expressions of PPA1796, PPA2105, and *hly* in *C. acnes* [37]. Alizarin has also been reported to downregulate two adhesin and two invasion-related genes [1] and PPA1715 but not to affect the expressions of two other adhesins (PPA1983 and PPA1906) [38], which suggests that alizarin might inhibit the attachment to and the invasion of host tissue. Our porcine skin results also support this suggestion (Figure 7).

Alizarin also significantly suppressed the expressions of various virulence-related genes, such as *btuR* (cobinamide adenosyltransferase), PPA0149 (glycosyltransferase), PPA0349 (polysaccharide capsule biosynthesis related), PPA1035 (hydrolase), PPA1098 (heat shock protein), PPA1222 (polyprenol monophosphomannose synthase), and PPA2139 (cutinase), and somewhat surprisingly, upregulated the antioxidant gene *roxP* and hemolysin (*tly*). RoxP has been reported to promote skin colonization by *C. acnes* ex vivo [39] and hemolysin is considered to function as a positive role for the pathogenesis of acne vulgaris. These results suggest alizarin may regulate the virulence of *C. acnes* and that more comprehensive transcriptomic and phenotypic studies are required on the topic.

## 5. Conclusions

In the present study, alizarin prevented biofilm development by *C. acnes*, *S. aureus*, and *C. albicans* in single- and three-species biofilm models under anaerobic conditions. When co-administered with antibiotic treatments, alizarin enhanced antibiofilm activities (Figure S1). Furthermore, alizarin eliminated *C. acnes* biofilms from porcine skin (Figure 7). These results propose that alizarin has potential use as an antibiofilm means and antibiotic adjuvant. In addition, we suggest additional studies be undertaken to explore the antibiofilm activities of other anthraquinone derivatives against *C. acnes* biofilms and multispecies biofilms and the structure activity relationships of anthraquinones. Despite the antibiofilm activities of alizarin against several microbes, the low water solubility limits its further applications. To solve the solubility issue of alizarin, drug delivery systems, such as polymeric nanocapsules, bipolymeric-coated alizarin nanocarriers [40], hydrogels [41], and liposomes can be utilized. Furthermore, the antibiofilm efficacy of alizarin should be confirmed in in-vivo animal models or human skin.

## Data Availability

All data relevant to the publication are included.

## Supplementary Materials

The following supporting information can be downloaded at: https://www.mdpi.com/article/10.3390/pharmaceutics14051047/s1, Figure S1: Combinatory antibiofilm activity of alizarin along with antibiotics against *C. acnes*; Table S1: qRT-PCR primer sequences used for *C. acnes*.

## Abbreviations

ATCC	American type culture collection
CFDA-SE	Carboxyfluorescein diacetate succinimidyl ester
CFU	Colony-forming unit
CLSM	Confocal laser scanning microscopy
CSH	Cell surface hydrophobicity
DMSO	Dimethyl sulfoxide
EPS	Extracellular polymeric substances
KCCM	Korean culture center of microorganisms
LUT	Lookup table
MIC	Minimum inhibitory concentration
LB	Lysogeny broth
PBS	Phosphate-buffered saline
PDB	Potato dextrose broth
qRT-PCR	Quantitative real-time reverse transcription polymerase chain reaction
RCM	Reinforced clostridium media
SEM	Scanning electron microscopy
