# Supplementary material for: Phytopigment Alizarin Inhibits Multispecies Biofilm Development by Cutibacterium acnes, Staphylococcus aureus, and Candida albicans
Source: Pharmaceutics. 2022 May 12;14(5):1047. doi: 10.3390/pharmaceutics14051047 (PMC9143108; doi:10.3390/pharmaceutics14051047)
Supplement: Supplementary file 1 [file pharmaceutics-14-01047-s001.zip › pharmaceutics-1644957-supplementary.pdf]

pharmaceutics

MDPI

# Supplementary Materials

# Phytopigment alizarin inhibits multispecies biofilm development by *Cutibacterium acnes*, *Staphylococcus aureus*, and *Candida albicans*

**Jin-Hyung Lee [1,†], Yong-Guy Kim [1,†], Sunyoung Park [1], Liangbin Hu [2] and Jintae Lee [1,*]**

[1] School of Chemical Engineering, Yeungnam University, 280 Daehak-Ro, Gyeongsan, 38541, Republic of Korea; jinhlee@ynu.ac.kr (J.-H.L.); yongguy7@ynu.ac.kr (Y.-G.K.); sunyong3142@ynu.ac.kr (S.P.)
[2] School of Food & Biological Engineering, Shaanxi University of Science and Technology, People's Republic of China; 4437@sust.edu.cn (L.H.)
* Correspondence: jtlee@ynu.ac.kr; Tel.: +82-53-810-2533, Fax: +82-53-810-4631
† Those authors contributed equally to this work.

**Table S1. qRT-PCR primer sequences used for *C. acnes*.**

| Gene | Description | Primer |
| --- | --- | --- |
| **PPA1761** | Lipase | Forward 5'-CCT CAA GGT GTC TGG TCA TC-3' |
| | | Reverse 5'-GAC TTC GTC GTT TTT CCA GA-3' |
| **PPA1796** | Lipase | Forward 5'-CTC AAG GTT CGT GAA CGA GT-3' |
| | | Reverse 5'-GCC ATA GAG CTC CTT GTT GA-3' |
| **PPA2105** | Lipase | Forward 5'-GAT TTC CTT AGC ACG TGG AG-3' |
| | | Reverse 5'-GAT GAC GGT GTA GGC GAT AC-3' |
| **PPA1035** | Alpha/beta hydrolase, lipase | Forward 5′-TAT CGA TCA TGG GTG ATT CC-3' |
| | | Reverse 5′-ATA GAG GGC GAG GAC TGA CT-3' |
| **PPA380** | Hyaluronate lyase | Forward 5′-CGC TCT GAA GGA TTC GTC-3′ |
| | | Reverse 5′-GTC GTG CAG GAT ACA CAT GA-3′ |
| *hly* | Hyaluronate lyase | Forward 5′-CAA CAT CGC CGT GTT TAT TG-3′ |
| | | Reverse 5′-CCC ATG ACG ACG TAG AGG AT-3′ |
| **PPA0721** | Invasion-associated protein | Forward 5′-AGA AGA AGG AAC AGG AGG CC-3′ |
| | | Reverse 5′-TCA GGA ATC GGC ACT TGA CT-3′ |
| **PPA1962** | Invasion-associated protein | Forward 5′-TTC AGT AAA GGC CCG AGT GT-3′ |
| | | Reverse 5′-ACA GGT AGT GGT GGT CGA TG-3′ |
| **PPA1983** | Adhesins (Thrombospondin type 3 repeats, PKD, SEST domains) | Forward 5′-AAG GGG ATT ACG GCT GTC AA-3′ |
| | | Reverse 5′-CAG CCT GGT CTT TGT GGT TC-3′ |
| **PPA1906** | | Forward 5′-AAG GGG ATT ACG GCT GTC AA-3′ |

| | | |
|---|---|---|
| | Adhesins (Thrombospondin type 3 repeats, PKD, SEST domains), PTRPs/host-interacting factors | Reverse 5′-CAG CCT GGT CTT TGT GGT TC-3′ |
| **PPA1715** | PTRPs (Proline-Threonine Repetitive Protein, recognizing human fibrinogen) /host-interacting factors | Forward 5′-GTT CAA GCT GTC CCA CAA GG-3′<br>Reverse 5′-TTG CTA TCG GTG AGG GTC TC-3′ |
| **PPA2127** | | Forward 5′-TCA CTG ATG CGA AGA TCC AG-3′<br>Reverse 5′-TTG CCA ACT TCT TGT CAA CG-3′ |
| ***luxS*** | *S*-ribosylhomocysteine lyase | Forward 5′-AGC CCA ACA AGG AAC ATC TG-3′<br>Reverse 5′-TGT TGA GCT TGA GGA TGT CG-3′ |
| **PPA0149** | Glycosyltransferase | Forward 5'-AGT ACA TGG CTT CCC GAG TG-3'<br>Reverse 5'-CTT GGG ACT CGA AGT TGA GC-3' |
| ***btuR*** | Cobinamide/cobalamin adenosyltransferase | Forward 5'-GGA AGA TGC TCT TCG GGC GCT-3'<br>Reverse 5'-GCC TCA GGG TTC TCC GCA GC-3' |
| ***cbiL*** | Precorrin-2 C(20)-methyltransferase | Forward 5'-GCG CGA GGC AGA CGT GAT CC-3'<br>Reverse 5'-GAC ACC GGA CCT CTC CCG CA-3' |
| ***roxP*** | Radical oxygenase | Forward 5'-GCA TCT AGC CCT CTC ACC AT-3'<br>Reverse 5'-CTG AGA GTC CGG TAG GTG GT-3' |
| ***tly*** | Lysis of red blood cells | Forward 5'-CAG GAC GTG ATG GCA ATG CGA-3'<br>Reverse 5'-TCG TTC ACA AGA CCA CAG TAG C-3' |
| **PPA0349** | Polysaccharide capsule biosynthesis | Forward 5′-CTT CTT CGT CGA CCA GTT CC-3'<br>Reverse 5′-TCA GCT GTC TCG TCA ACA CC-3' |
| **PPA1098** | DnaK, Hsp70 family protein | Forward 5′-TGG ATC TGG GAG TCG ACC TA-3'<br>Reverse 5′-TTA GCA CCG TTG AGC AGA GA-3' |
| **PPA2139** | Cutinase | Forward 5′-CGA GAA TGG ACT GTG CTT CG-3'<br>Reverse 5′-GTT GAT GGC ATC GTG GAG TC-3' |
| **PPA1222** | Polyprenol monophosphomannose synthase | Forward 5′-ATG ACG CCC TAG TCG AAA TG-3'<br>Reverse 5′-ATG AGT TCG CGG TAC TTT GG-3' |
| ***16s rRNA*** | 16S ribosomal RNA (House keeping gene) | Forward 5'-GGG GCT TAA CCC TGA GCG TGC -3'<br>Reverse 5'-TTC GCT CCC CAC GCT TTC GC-3' |

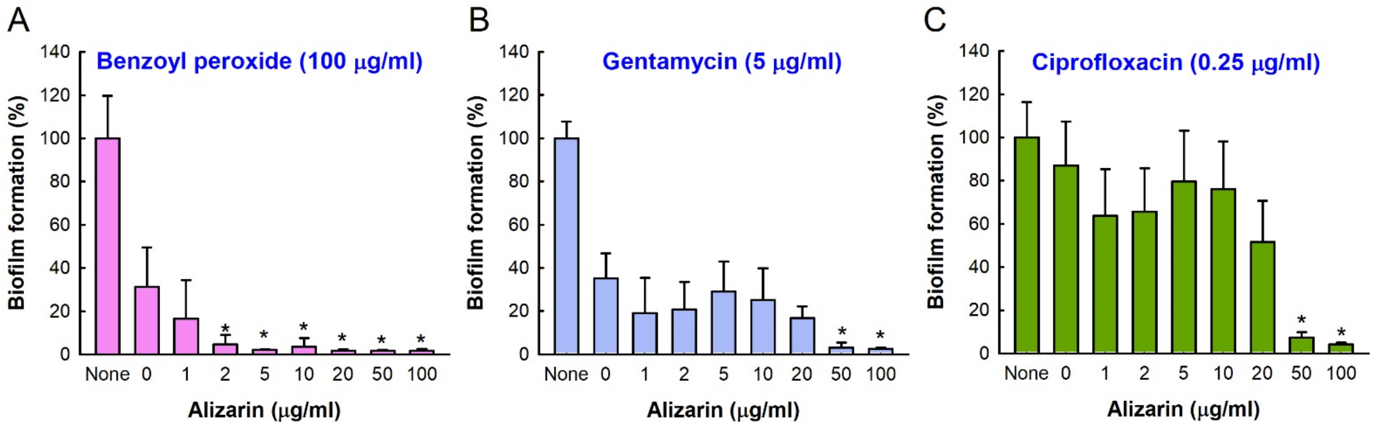


**Figure S1. Combinatory antibiofilm activity of alizarin along with antibiotics against *C. acnes*.** Biofilm inhibitions by benzoyl peroxide at 100 μg/ml (A), gentamycin at 5 μg/ml (B), and ciprofloxacin 0.25 μg/ml (C) with alizarin (0, 1, 2, 5, 10, 20, 50, and 100 μg/ml) were measured after incubation for 6 days in 96-well plates at 37°C under anaerobic conditions. Error bars indicate standard deviations. *, $P < 0.05$ *vs.* non-treated controls.
